# Salivary stress biomarkers are increased, independently of maternal welfare status, in lactating Iberian piglets with lower postnatal growth

**DOI:** 10.3389/fvets.2026.1772189

**Published:** 2026-02-11

**Authors:** Natalia Yeste-Vizcaino, Beatriz Isabel, José Joaquín Cerón, Alberto Muñoz-Prieto, Antonio Gonzalez-Bulnes

**Affiliations:** 1 Cuarte S.L., Grupo Jorge, Zaragoza, Spain; 2 Faculty of Veterinary Medicine, Universidad Autónoma de Barcelona, UAB, Barcelona, Spain; 3 Faculty of Veterinary Medicine, Universidad Complutense de Madrid, UCM, Madrid, Spain; 4 Interdisciplinary Laboratory of Clinical Analysis (Interlab-UMU), Veterinary School, University of Murcia, Murcia, Spain; 5 Faculty of Veterinary Sciences, Universidad CEU-Cardenal Herrera, CEU Universities, Valencia, Spain; 6 Mensoft Consultores S.L. Madrid, Madrid, Spain

**Keywords:** biomarkers, health, Iberian-breed, saliva, swine, welfare

## Abstract

Iberian pigs represent a small but economically significant segment of the Spanish swine sector, valued for the high quality of their products and the strong welfare-friendly image associated with their production systems. As management practices increasingly transition toward more intensive housing, reliable assessment of stress and welfare becomes essential. This study presents the first comprehensive characterization of salivary stress biomarkers—cortisol, *α*-amylase (sAA), butyrylcholinesterase (BChE), and total esterase activity (TEA)—in 68 Iberian sows across key reproductive stages (early, mid-, and late- gestation; mid-lactation; and weaning) and their relationship with piglet health and performance. Sows showed the highest biomarker levels early in gestation, with values decreasing as pregnancy and lactation progressed. Handling-intensive events elicited pronounced stress responses, especially in primiparous sows. No associations were detected between salivary biomarkers and sow body weight or back-fat depth. Piglets exhibited higher biomarker values at weaning than sows, reflecting acute handling stress. Sex and maternal parity did not significantly affect piglet biomarkers. Lower postnatal body weight was associated with elevated cortisol, sAA, and TEA, indicating increased physiological stress in lighter piglets. These findings validate salivary biomarkers as practical, non-invasive indicators of welfare in Iberian pigs and provide essential reference patterns to support improved management and welfare assessment in this breed.

## 1 Introduction

The Iberian pork, although only represents 6% of the total swine production in Spain (1), have gained market relevance worldwide due to their widely recognized nutritional and sensorial quality, both in fresh meat (2, 3) and dry-cured products ham, loin, salami, bacon, and others (4). Thus, aiming to increase its productive efficiency, the traditional extensive management of the breed is changing to intensive systems like those used in lean commercial pigs.

However, such change may compromise the physiology, metabolism and behavior of the Iberian breed, which are shaped by centuries of exposure to extensive semi-feral free-ranging conditions (5, 6) and thus the productive efficiency of the pigs. Intensive systems, adopted to meet rising demand for animal products, are recognized sources of stress and welfare reduction (7). Hence, intensification of the management may also compromise the positive perception of the Iberian pork, as associated with sustainable practices and high-welfare husbandry, by the consumer. Consumers are increasingly attentive to production systems, environmental impact, and animal welfare; these attributes enhance the added value of the product and the customer willingness to pay for Iberian products (8, 9). Consequently, the equilibrium between welfare and stress is critical to Iberian pig production, due to their economic impact and public relevance.

Evaluation of animal emotions is challenging; thus, most of the initiatives propose multidimensional assessment protocols based on behavior, productivity, and pathological and physiological parameters (10); however, these schemes lack precision and objectivity. Assessment of biomarkers (e.g.: cortisol) gains in objectivity but no single biomarker currently fulfils ideal criteria (reliability, specificity, sensitivity, predictive value, low invasiveness, and easiness of measuring (11)). Consequently, combinations of biomarkers are increasingly used to evaluate welfare, facilitate husbandry improvements, support disease prevention, and advance understanding of stress-related molecular processes. Assessment of biomarkers is also increasingly applied in saliva instead of blood because saliva sampling, being non-invasive and painless, improves welfare and allows repeated sampling without specialized personnel. These features are particularly important in pigs, in which blood sampling induces substantial stress (12).

Cortisol is the most used salivary stress marker in pigs since stress activates the hypothalamic–pituitary–adrenal (HPA) axis, leading to increases glucocorticoid secretion (particularly, cortisol). However, its application is weakened by a high inter- and intra-individual variability determined by genetics, circadian rhythm, age, and sex (13, 14). Salivary *α*-amylase (sAA) is increasingly used since reflects activation of the sympathetic-adrenal-medullary (SAM) axis and correlates with plasma catecholamines (15). However, its response to acute stress is highly variable and some pigs may show no increases after stress induction (16, 17). Total esterase activity (TEA; comprising enzymes such as lipase, carbonic anhydrase VI, and predominantly butyrylcholinesterase, BChE), is abundant in pig saliva and serves as a robust marker of acute stress, although its components respond differently to stressors and pathological conditions (18). However, despite the potential usefulness of these salivary biomarkers for assessing welfare status, there are no previous studies, to the best of our knowledge, on reference range values and modulating biological factors of salivary biomarkers in the Iberian pig.

In intensive swine breeding, productive stages such as gestation, farrowing, and lactation may be associated with increased stress (19, 20). We hypothesize that this stress could be reflected in variations in salivary welfare-related analytes. Therefore, the objectives of the present study were: (a) to characterize salivary stress biomarkers, particularly cortisol concentrations and the activities of *α*-amylase, butyrylcholinesterase, and total esterases in Iberian sows and their piglets; (b) to determine the potential influence of sow body condition and production stage (from the onset of gestation to the end of lactation); and (c) to assess possible relationships between salivary biomarkers and the productive performance of both sows and their piglets.

## 2 Materials and methods

### 2.1 Ethical statement

The study was conducted on a commercial farm (La Frondosa, Garrapinillos. Zaragoza, Spain) in accordance with Spanish Policy for Animal Protection RD53/2013, which aligns with the European Union Directive 2010/63/EU on the protection of animals used for scientific purposes. The Animal Research Ethics Committee of Universidad Complutense de Madrid reviewed and approved all experimental procedures (CEEAH2788M2).

### 2.2 Animals and management

This study involved a total of 68 Iberian sows and their live-born piglets (*n* = 543). At starting, the trial involved 70 sows which were found pregnant after an ultrasonographic assessment performed 35 days post-insemination; however, two sows were discarded by health issues during lactation. The management of sows and their offspring followed standard farm practices, with the animals being housed indoors under controlled temperature. Both sows and piglets underwent daily general veterinary clinical examination on the farm. The parameters assessed included the overall appearance of each animal and the recording of any clinical signs of disease or abnormal behavior.

On Day 35 of pregnancy, all the sows were grouped in lots (*n* = 10) and moved to collective pens where remained until Day 105 of pregnancy, when they were moved to farrowing rooms. All sows had *ad libitum* access to water and were fed once daily (07:00 h) with a standard barley-based diet (21), following a reference feed intake curve providing 1.6 kg of feed from Day 35 to Day 85 and 1.8 kg of feed from Day 85 to Day 105. The feed quantity could be manually adjusted in the feeders by farm personnel based on the previous day’s feed intake.

On Day 105 of pregnancy, around one week before delivery, each group of 10 sows was kept together and moved to a farrowing room consisting of 10 traditional farrowing crates equipped with electronic sow feeders (Gestal SOLO+, Jyga Technologies Inc., Quebec, Canada). Sows had *ad libitum* access to water and were fed six rations per day (05:00, 08:00, 11:00, 14:00, 17:00, and 20:00) with a standard barley-based diet (21), following a reference feed intake curve, which increased gradually from the day of farrowing until Day 13 and remained afterwards at a plateau until weaning at Day 25 after farrowing. The feed amount was automatically adjusted by the device, allowing each sow to consume up to 20% more feed per meal.

At farrowing, productive data (total number of live-born and stillborn piglets) were recorded for each sow. At birth, the sex and individual weight of every piglet (both live-born and stillborn) were documented. Immediately thereafter, live-born piglets (*n* = 543) were identified using electronic ear tags for subsequent monitoring. At 48 h of age, these piglets were cross-fostered to achieve approximately eight piglets per sow with similar birth weights. Creep feed was offered to all piglets from 14 days of age. At 25 days of age, piglets were weaned and transferred to a nursery farm, where they remained for 42 days (until approximately 67 days of age). Productive traits (changes in body weight) were assessed in all piglets during the suckling and nursery periods, while salivary biomarkers of health and welfare were analyzed in a subset of 136 piglets.

### 2.3 Changes in body-weight and -condition of sows

Body-weight and -condition (back-fat depth) were determined in all the sows during gestation and lactation. Specifically, the sows were weighed at five time-points: three during gestation (day 35 or first third of gestation, day 70 or second third of gestation, and day 105 or end of gestation) and two during lactation (day of parturition and day of weaning).

Back-fat depth (BFD) was measured at P2 point, which lies on the right side of the animal at 4 cm from the midline and transversal to the head of the last rib, using ultrasound equipment fitted to a multifrequency linear array probe (ProVetScan SF2 Wireless scanner, NewVetec, Leon, Spain). Specifically, BFD of the sows was measured at four time-points: three during gestation (day 35 or first third of gestation, day 70 or second third of gestation, and day 105 or end of gestation) and day of weaning.

Initial and final body-weight (BW) and BFD of the sows during both phases of gestation and lactation were used to calculate gain in body-weight and condition during gestation (BWG and BFDG, respectively), and losses in body-weight and condition during lactation (BWL and BFDL, respectively).

### 2.4 Piglets growth

Piglets were individually weighted at birth, at 14 days of age (when creep feeding was offered), at weaning (around 25 days of age), and at the end of the nursery period (around 68 days of age). Average daily weight gain (ADWG) was individually determined for all piglets, using the formula ([final weight—initial weight] / number of days elapsed) for the intervals of 0–14, 0–25, and 25–68 days of age.

### 2.5 Saliva collection and processing

Assessment of health/welfare saliva biomarkers in sows was performed at five time-points; three during pregnancy (Day 35 or first third of gestation, Day 70 or second third of gestation, and Day 105 or end of gestation) and two during lactation (14 and 25 days after farrowing).

Assessment of health/welfare saliva biomarkers in piglets, and possible relationships between traits of sows and piglets, was performed on the day of weaning, when saliva samples were collected in the subset of 136 piglets; among a representative in size and behavior population (*n* = 2) in each litter, one male and one female from each litter, avoiding the smallest and largest piglets.

Saliva samples were obtained in both sows and piglets by using Salivette® tubes (Sarstedt, Nümbrecht, Germany) and a polypropylene sponge. The sponge, attached to a thin and flexible metal rod, was offered to be chewed by either sows or piglets for around 30 s, taking advantage of their innate curiosity and thus minimizing handling-induced stress. Once fulfilled with saliva, the sponges were introduced in the Salivette^®^ tubes and centrifuged for 10 min at 3,000 × g. At once, the resulting saliva sample (around 0.5–1.0 mL in volume) was transferred to 1.5 mL polypropylene tubes and stored at −80 °C until analyzed.

### 2.6 Saliva analysis

All the salivary biomarkers assessed in the present study (concentration of cortisol and activity of *α*-amylase, total esterase, and butyrylcholinesterase) were measured using an automated analyzer (Olympus AU400, Olympus Diagnostica GmbH, Ennis, Ireland), at an absorbance at 405 nm.

In brief, salivary cortisol concentration was measured using an automated chemiluminescent enzyme immunoassay (Immulite 1,000 Cortisol; Siemens Medical Solutions Diagnostics), previously validated for pig saliva (22). Salivary *α*-amylase (sAA) activity was measured using a commercial colorimetric kit (Alpha-Amylase; Beckman Coulter Inc., Fullerton, CA, USA), previously validated for pigs (17) following the method of the International Federation of Clinical Chemistry and Laboratory Medicine (23). Total esterase (TEA) activity was measured using a method based on the hydrolysis of the substrate 4-nitrophenyl acetate (4-NA; Sigma-Aldrich Co., St. Louis, MO, USA) by salivary esterases, as described in human saliva by Tecles et al. (24) and afterward validated for pig saliva (25). Salivary butyrylcholinesterase (BChE) activity was measured using the Ellman method, originally developed for whole blood (26) and validated with minor adaptations for saliva samples (18).

### 2.7 Statistical analysis

Statistical analyses were performed using SPSS 26.0 (IBM, Chicago, IL, USA). Normality of data was assessed using the Kolmogorov–Smirnov test and, when needed, data were log-transformed to correct distribution and allow the use of parametric statistics. For sow data, a repeated-measures general linear model (GLM) was used to compare values across the five sampling time points of the production cycle. In the factorial model, each sow was included as the experimental unit, sampling time points as intra-subject effect, and the number of parity (primiparous or multiparous) as inter-subject effect. All piglet variables were analysed using UNIANOVA with Bonferroni adjustment. Each piglet was included as the experimental unit, with fixed effects including sex (female or male), the sow’s number of parity (primiparous or multiparous), and the interaction between them. Potential correlations between variables were analysed using Pearson’s method for normally distributed variables and Spearman’s method for non-normally distributed variables. Correlations were considered from *R* > 0.500 and a significance of *p* < 0.05. Results are presented as mean ± standard error of the mean (SEM), with *p* < 0.05 representing statistical significance and 0.05 < *p* < 0.10 denoting a trend.

## 3 Results

### 3.1 Assessment of salivary biomarkers in sows during pregnancy and lactation

**Figure 1:**
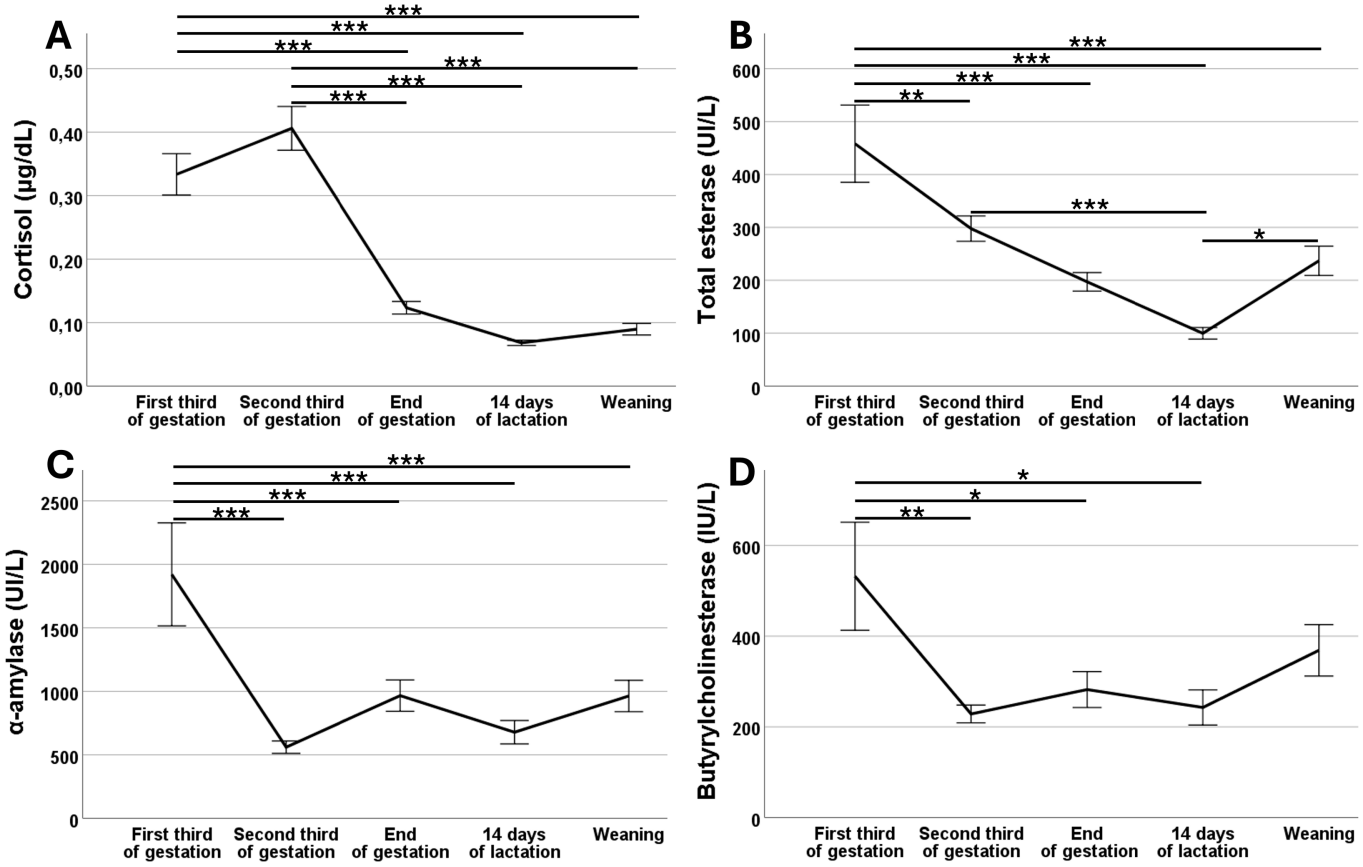
Changes over time (mean ± S.E.M.) in salivary concentration of cortisol **(A)** and activity of total esterase **(B)**, α-amylase **(C)**, butyrylcholinesterase **(D)** during pregnancy and lactation of Iberian sows. Asterisks indicate significant differences (*p < 0.05; **p < 0.01; ***p < 0.001).

Mean values for salivary concentration of cortisol and activity of butyrylcholinesterase (BChE), total esterases (TEA), and *α*-amylase (sAA) in the sows are reported in Figure 1 and Table 1.

**Table 1:** Mean values (± S.E.M.) of salivary concentration of cortisol (μg/dL) and activity (IU/L) of total esterase (TEA), α-amylase (sAA) and butyrylcholinesterase (BchE) during pregnancy and lactation of primiparous and multiparous Iberian sows.

Timing	Biomarker	Primiparous	Multiparous
First third of gestation	Cortisol	0.33 ± 0.04	0.33 ± 0.05
	TEA	519.43 ± 125.78	404.63 ± 83.24
	sAA	2867.14 ± 682.76	975.00 ± 282.67*
	BChE	809.36 ± 220.50	273.20 ± 53.21**
Second third of gestation	Cortisol	0.42 ± 0.04	0.37 ± 0.06
	TEA	301.06 ± 27.19	288.00 ± 51.46
	sAA	564.96 ± 54.57	545.63 ± 109.91
	BChE	209.11 ± 15.41	288.80 ± 64.27
End of gestation	Cortisol	0.11 ± 0.01	0.15 ± 0.03
	TEA	191.80 ± 20.59	212.65 ± 33.93
	sAA	1039.31 ± 150.40	714.64 ± 171.17
	BChE	285.25 ± 50.27	272.60 ± 45.22
14 days of lactation	Cortisol	0.07 ± 0.01	0.07 ± 0.01
	TEA	122.20 ± 18.87	79.94 ± 10.49
	sAA	676.25 ± 119.98	679.24 ± 134.98
	BChE	300.69 ± 61.69	195.56 ± 47.71
Weaning	Cortisol	0.10 ± 0.01	0.06 ± 0.00*
	TEA	258.50 ± 36.08	173.12 ± 19.43
	sAA	1120.66 ± 151.18	439.00 ± 98.26**
	BChE	335.70 ± 65.15	472.19 ± 115.49

Asterisks denote significant differences between primiparous and multiparous sows in each production phase (**p < 0.01; *p < 0.05).

There were no major significant effects of the parity number (primiparous vs. multiparous) on these biomarkers during gestation and lactation. Overall, cortisol levels were highest during the first two samplings at gestation (Days 35 and 70 of pregnancy) than at the end of pregnancy and the entire lactation (*p* < 0.001). Activity of TEA, sAA and BChE was highest at Day 35 to decrease thereafter, during pregnancy and lactation, and increase again on the day of weaning.

However, significant differences in salivary biomarkers were observed between primiparous and multiparous sows when assessing the 2 days with the most intensive handling: the day of pregnancy diagnosis and grouping in pregnancy pens (Day 35 of pregnancy) and the day of weaning (Day 25 of lactation). On Day 35 of pregnancy, primiparous sows exhibited higher activity than multiparous sows for both BChE (*p* < 0.01) and sAA (*p* < 0.05). On Day 25 of lactation, primiparous sows showed higher cortisol levels (*p* = 0.021) and greater sAA activity (*p* = 0.003) than multiparous sows.

### 3.2 Relationships between salivary biomarkers and body-weight and condition of sows

Mean values for body-weight and back-fat depth (BFD) of sows over gestation and lactation, distributed by number of parity are reported in Table 2. At the beginning of the study, both groups of sows had similar body-weight (*p* > 0.05), but the primiparous sows had lower BFD (*p* = 0.007). However, these differences disappeared over time of pregnancy and the sows finished pregnancy in similar conditions. Afterwards, there were again significant differences in the evolution of BW during lactation (BWL, *p* < 0.001) and, the primiparous sows showed a lower BW at weaning than multiparous sows (*p* = 0.01).

**Table 2:** Mean values (± S.E.M.) for body-weight (BW, kg) backfat depth (BFD, mm), and gain or losses in body-weight and condition during gestation (BWG and BFDG) and lactation (BWL and BFDL) of primiparous and multiparous Iberian sows.

Trait	Primiparous	Multiparous
BW at first third of gestation	160.51 ± 1.82	158.75 ± 3.77
BFD at first third of gestation	33.07 ± 1.05	38.67 ± 1.35**
BW at second third of gestation	165.35 ± 2.02	168.35 ± 3.51
BFD at second third of gestation	38.21 ± 1.01	40.25 ± 1.27
BW at the end of gestation	180.00 ± 2.20	177.24 ± 3.98
BFD at the end of gestation	38.40 ± 1.02	38.38 ± 1.33
BWG	19.35 ± 1.42	19.63 ± 1.86
BFDG	12.42 ± 1.09	23.90 ± 1.40***
BW at farrowing	170.16 ± 2.07	169.41 ± 3.41
BW at weaning	159.98 ± 2.12	171.12 ± 3.41**
BFD at weaning	29.39 ± 0.86	28.25 ± 0.96
BWL	10.18 ± 1.48	−1.71 ± 1.58***
BFDL	9.00 ± 0.73	10.12 ± 1.01

Asterisks denote significant differences between primiparous and multiparous sows (***p < 0.001; **p < 0.01).

There were not found any relationships among BW and BFD in any of the sampling points, and thus in the changes during pregnancy and lactation (BWG, BFDG, BWL or BFDL), with either the concentration of cortisol or the activity of butyrylcholinesterase (BChE), total esterases (TEA), or *α*-amylase (sAA) in the saliva of sows.

### 3.3 Assessment of salivary biomarkers in piglets at weaning

Mean values for salivary concentration of cortisol and activity of butyrylcholinesterase (BChE), total esterases (TEA), and α-amylase (sAA) in weaned piglets, distributed by sex of the piglet and number of parity of the sow, are reported in Table 3. Neither piglet’s sex nor sow’s parity showed significant effects on salivary biomarkers at weaning. However, a significant interaction between piglet’s sex and sow’s parity was observed for BChE activity (*p* < 0.05), with a trend for lower BChE activity in male piglets than in female piglets from primiparous sows (*p* = 0.057, pairwise comparison) and in male piglets from primiparous sows than in males from multiparous sows (*p* = 0.053).

**Table 3:** Mean values (± S.E.M.) distributed by sex of the piglet and number of parity of the sow, of salivary concentration of cortisol (μg/dL) and activity (IU/L) of total esterase (TEA), α-amylase (sAA) and butyrylcholinesterase (BchE) in Iberian piglets at weaning.

Biomarker	Sex	Primiparous	Multiparous	Parity	Sex	Interaction
Cortisol	Female	0.11 ± 0.01	0.13 ± 0.01	0.495	0.656	0.090
	Male	0.12 ± 0.01	0.11 ± 0.01			
TEA	Female	1737.60 ± 231.27	1286.21 ± 136.93	0.474	0.865	0.184
	Male	1551.33 ± 188.77	1594.10 ± 193.11			
sAA	Female	26222.22 ± 3978.55	23059.74 ± 3048.83	0.279	0.322	0.696
	Male	24508.65 ± 3171.32	29106.00 ± 3688.08			
BChE	Female	3199.12 ± 417.20	2348.89 ± 298.02	0.498	0.444	**0.041**
	Male	2300.26 ± 337.72	3230.81 ± 439.68			

Statistical significance is indicated by bold values.

**Figure 2:**
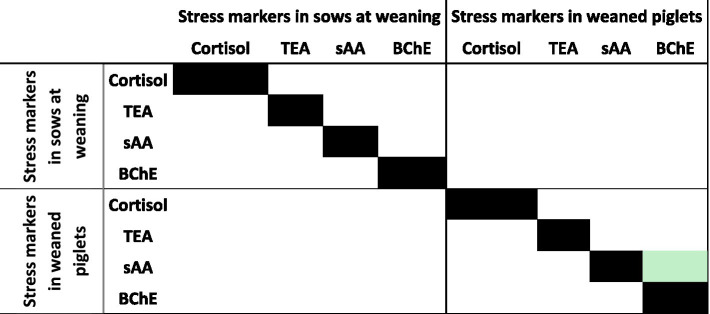
Heatmap representation of correlations among stress markers in sows and piglets at weaning. Significant correlations (*p* < 0.05) are shown according to the *R*-value (Light green *R* > 0.50).

There was also found a positive relationship of BChE activity with sAA activities (R^2^ = 0.678, *p* < 0.001), as depicted in Figure 2. However, assessment of possible relationships among biomarkers in sows and their piglets showed no significant correlations at weaning.

### 3.4 Relationships between salivary biomarkers and postnatal development in piglets

Mean values for body-weight of piglets over lactation and nursery, distributed by sex of the piglet and number of parity of the sow, are reported in Table 4. A higher birth weight was associated with higher body-weights throughout postnatal development (14 days of lactation, weaning and nursery period; *p* < 0.001).

**Table 4:** Mean values (± S.E.M.), for body-weight (BW, kg) and Average Daily Weight Gain (ADWG, kg/day) of Iberian piglets, distributed by sex of the piglet and number of parity of the sow, during lactation, and nursery.

Trait	Sex	Primiparous	Multiparous	Cycle	Sex	Interaction
BW at birth	Female	1.44 ± 0.05	1.32 ± 0.03	**0.003**	**0.024**	0.754
	Male	1.56 ± 0.06	1.41 ± 0.04			
BW at 14 days	Female	4.63 ± 0.22	4.32 ± 0.17	0.320	0.442	0.488
	Male	4.41 ± 0.20	4.49 ± 0.19			
ADWG at 14 days	Female	0.20 ± 0.01	0.17 ± 0.01	0.119	0.397	0.087
	Male	0.18 ± 0.01	0.19 ± 0.01			
BW at weaning	Female	7.21 ± 0.36	7.01 ± 0.25	0.675	0.117	0.716
	Male	7.22 ± 0.29	7.64 ± 0.25			
ADWG at weaning	Female	0.19 ± 0.01	0.18 ± 0.01	0.478	0.066	0.288
	Male	0.19 ± 0.01	0.21 ± 0.01			
BW at transition	Female	25.19 ± 0.93	25.83 ± 0.89	0.482	**0.009**	0.992
	Male	27.63 ± 0.84	28.29 ± 0.98			
ADWG at transition	Female	0.41 ± 0.01	0.43 ± 0.02	0.271	**0.005**	0.890
	Male	0.46 ± 0.01	0.48 ± 0.02			

Statistical significance is indicated by bold values.

Piglet sex significantly affected birth weight and both body-weight and ADWG at the end of the nursery period, with males being heavier than females.

**Figure 3:**
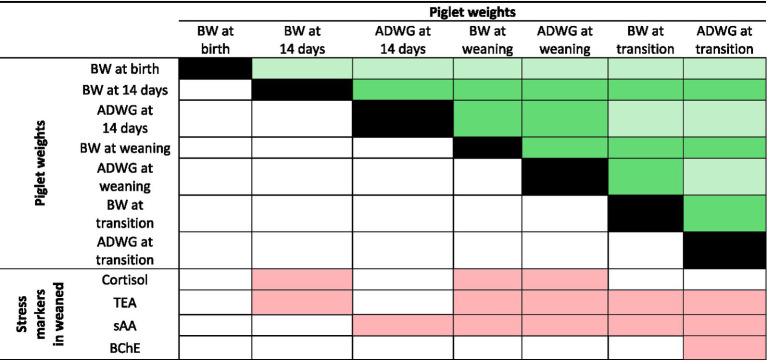
Heatmap representation of correlations among changes in body-weight and stress markers in piglets. Significant correlations (*p* < 0.05) are shown according to the *R* value (Dark green *R* > 0.75; Light green *R* > 0.50; Light red *R* < −0.50).

Assessment of possible relationships between changes in body-weight and stress biomarkers of piglets (Figure 3) showed that body-weight at 14, 25, and 67 days of age (i.e.: lactation and nursery) was negatively related to cortisol concentration and activities of TEA and sAA at weaning. In other words, independently of birth-weight and sex, piglets reaching higher body-weights during lactation showed reduced stress biomarkers (i.e.: lower enzymatic activity and cortisol concentrations).

## 4 Discussion

The present trial represents, to the best of our knowledge, the first study measuring and characterizing changes over time of several stress biomarkers (concentrations of cortisol and activities of *α*-amylase, butyrylcholinesterase and total esterases) in saliva samples of Iberian pigs allowing to increase the knowledge about the non-invasive reliable indicators of animal welfare in this breed. Our results indicate that such salivary biomarkers are modulated by the number of parity of the sow (primiparous or multiparous) and its management throughout different productive stages (from the onset of gestation to the end of lactation), independently of its body-weight and -condition. In the case of piglets, salivary stress biomarkers were found to be increased, independently of sex and maternal welfare status, in those with lower postnatal growth.

Saliva sampling in both sows and their piglets was non-invasive, simple, cost-effective and reproducible. The use of swabs minimized the stress in the animals, since it was not necessary any management or immobilization, and took advantage of their innate curiosity and exploratory behavior. Management and storage of samples was also easy to perform and, afterwards, the laboratory assays and procedures showed high repeatability and linearity and were easily automated, which mean significant technical advantages.

Assessment of the biomarkers in the sows included three representative sampling time-points during pregnancy (Day 35 or first third of gestation, Day 70 or second third of gestation, and Day 105 or end of gestation) and two time-points during lactation (14 and 25 days after farrowing). Our results showed that all stress biomarkers of the sow were higher at the beginning of the study and decreased over time of pregnancy and lactation. There were no major significant effects of the parity number (primiparous vs. multiparous) or the body-weight or –condition of the sow on the values and changes over time of the salivary biomarkers. However, we found evidences of a higher stress in primiparous sows at the 2 days with the most intensive handling: the day of pregnancy diagnosis and grouping in pregnancy pens (Day 35 of pregnancy) and the day of weaning (Day 25 of lactation). Obviously, we can hypothesize a direct relationship between management and stress in non-experienced primiparous sows in agreement with previous studies (27).

Firstly, cortisol concentrations were significantly higher during gestation than during lactation, indicating an activation of the HPA axis in response to acute stress (28) probably due to formation of new groups in sows at gestation. Previous studies have reported similar findings, with group-housed sows exhibiting higher cortisol levels than individually housed sows but showing a decrease over time after transferring to farrowing pens (29, 30). Overall, these results support that formation of new groups in the gestation pens is a strong stressor in swine production, inducing aggression and social stress (31, 32) and that sows adapt to the new environment over time, leading to decreased levels of stress markers.

Assessment of salivary enzymes indicate that sAA activity was consistently higher in primiparous sows on days involving intensive handling, such as grouping in gestation pens and weaning, confirming the higher stress susceptibility of these females. However, sAA displayed high inter-individual variability, supporting previous studies (16, 17); a high variability which may reflect differences in the enzyme’s biological responsiveness to multiple stimuli, and which could limit its application as a reliable stress biomarker.

On the other hand, BChE activity remained similar across most production phases in the present study, except on the day when sows were grouped in the gestation pens, when primiparous sows exhibited higher activity than multiparous sows indicating that they are more susceptible to handling and movement stress. The role of BChE in salivary stress responses remains unclear (33), but a seminal study of Tecles et al. has shown dynamic changes in the BChE activity in response to acute stress (18). Analysis of TEA results supports these findings, as BChE is a major contributor to salivary TEA changes under acute stress (25). In our study, TEA activity peaked when sows were moved to confirmed gestation pens and decreased thereafter, resembling responses to acute restraint stress in other studies. Similar patterns were reported by Botia et al. (34), showing higher TEA levels in pigs subjected to more stressful handling compared to those managed less stressfully.

Our results also indicate no correlation between sAA and salivary cortisol, consistently with other studies (17, 35). This may be caused by different release mechanisms of both biomarkers, with sAA representing the adrenergic system and cortisol the HPA axis (36). Understanding the origins of salivary biomarkers helps elucidate mechanisms of stress response: cortisol originates from plasma (37), while BChE is produced in salivary glands and intestinal mucosal cells (26, 38), and sAA is secreted by salivary glands in response to SAM axis activation (15). On the other hand, there were found high correlations between BChE and sAA, activities, likely because of their shared salivary origin (15, 26).

Assessment of the biomarkers in the piglets at weaning showed significantly higher values than in sows, which likely reflect the handling method used to collect saliva, involving temporary immobilization, since our values resemble data obtained in pigs immediately after restraint (18, 25). Both BChE and sAA values in our piglets aligned with findings by Tecles et al. (39) in just weaned piglets of commercial lean strains. Stress biomarkers in the Iberian piglets were not related to parity number of the sow or with its own sex, consistently with prior works (39).

It is important to further emphasize the correlations identified between body weight and the stress-related biomarkers measured in piglets. In this sense, independently of sex and maternal welfare status, lower-weight lactating piglets showed higher cortisol levels as well as increased TEA and sAA activities, suggesting that they are more susceptible to stress at weaning. These results are like previously described in lean commercial strains in which it is well-known that birth-weight is a critical factor in pig production, health status, and overall welfare throughout the animal’s lifetime (40).

Genetic selection for higher prolificacy has resulted in reduced birth weights and, consequently, a higher prevalence of low-birth-weight (LBW) piglets. As reported previously, LBW piglets are generally unable to compensate for their early developmental disadvantage during postnatal growth, ultimately resulting in poorer performance and increased susceptibility to environmental and management-related stressors (41). Within the natural range of birth weights, LBW pigs are associated with changes in HPA axis function in later life. These alterations in the HPA axis may occur at the level of the hypothalamus, pituitary or adrenal gland itself. Although no direct evidence has demonstrated alterations in HPA function at the level of the hypothalamus and pituitary in juvenile pigs of LBW (42). Several studies have described an association between birth weight and adrenocortical function in subsequent early neonatal life. Specially, LBW piglets show heightened adrenal development (43), and increased cortisol production by adrenocortical cells, leading to elevated circulating cortisol (44). This endocrine profile contributes to an exaggerated stress response, which tends to persist throughout the animal’s life and may negatively affect welfare, resilience, and productive efficiency.

A a clear limitation of our study, is related to the fact that our assessment only included healthy animals in good condition and managed under optimal practice; hence, some results may vary in underfed or poorly managed animals with extreme low body-weight or condition. Nevertheless, the data reported here fulfils our aim of providing a reasonable approximation of the usefulness and the expected values when assessing salivary stress biomarkers in Iberian sows and piglets from commercial farms with normal rearing conditions and absence of disease outbreaks.

## 5 Conclusion

This study, the first one measuring a panel of several biomarkers (cortisol, BChE, TEA, and sAA) evaluated in the saliva of Iberian pigs, showed changes in sows depending of their productive cycle with increases in selected analytes in situations of stress. These salivary biomarkers are differentially modulated by the management of primiparous or multiparous Iberian sows, independently of its body-weight and -condition. Salivary stress biomarkers in the piglets were found to be increased, independently of sex and maternal welfare status, in lactating Iberian piglets with lower postnatal growth.

## Data Availability

The original contributions presented in the study are included in the article/supplementary material, further inquiries can be directed to the corresponding author.
